# The Structure of EMDR Therapy: A Guide for the Therapist

**DOI:** 10.3389/fpsyg.2021.660753

**Published:** 2021-05-25

**Authors:** Michael Hase

**Affiliations:** Lüneburger Zentrum für Stressmedizin, Lüneburg, Germany

**Keywords:** EMDR therapy, adaptive information processing, therapeutic relationship, case conceptualization, research, AIP model, treatment planning, education

## Abstract

Since the introduction of EMD by Dr. Shapiro in 1987, which led to the development of EMDR Therapy, clinical experiences and research contributed to a variety of protocols and procedures. While this dynamic evolution within EMDR Therapy is offering more options to treat a variety of patients suffering from various disorders, there is a greater risk of deviations from the core framework of this approach that would no longer be understood as EMDR Therapy. While research shows that following Shapiro’s standard protocols and procedural steps is important to achieve positive treatment effects, it seems prudent to define the core elements in EMDR Therapy beyond adherence to the standard protocol given the complexity of clinical demands in a variety of treatment contexts. The author proposes that best practices requires not only an adherence to the fidelity of the model, but a willingness to adapt the model in order to best meet the needs of our clients in a variety of clinical contexts. Defining the core elements that constitute EMDR Therapy offers both a structure that has been well established and offers a foundation from which clinical adaptations can be made that are within the realm of what is widely accepted as EMDR Therapy. Such a structure could also be used to define research as well as clinical applications. Additionally EMDR Therapy as a comprehensive psychotherapy approach implies that the therapeutic relationship is an important component and should be considered a core element of this methodology.

## Introduction

Eye Movement Desensitization and Reprocessing Therapy (EMDR) consists of a structured set of protocols and procedures based on the adaptive information processing (AIP) model (Shapiro and Laliotis, 2011). EMDR was introduced as EMD in 1987 (Shapiro, 1989) as a treatment for PTSD and was developed into the comprehensive therapy approach named EMDR Therapy over the course of time. Shapiro developed EMDR to be compatible with all major orientations to psychotherapy.

During the first years the concept of the eight phases, describing a comprehensive EMDR Therapy treatment, was developed. Even if the processing of inadequately processed and maladaptively encoded memories remains the primary focus of EMDR Therapy, a number of specialized protocols have been developed to address the needs of different clients suffering from a variety of disorders. For the scope of EMDR Therapy today see Valiente-Gomez et al. (2017). Some reflections on the AIP model and theory of pathogenic memories contribute to the theoretical foundation for the evolution of EMDR Therapy (Hase et al., 2017). An overview on the research on working mechanism in EMDR Therapy was recently provided by Landin-Romero et al. (2018). The neurophysiology behind bilateral stimulation has been researched to great extent (Pagani et al., 2017). Recently an animal model of bilateral stimulation by Eye movements (EM) has been described (Baek et al., 2019).

The theory currently used to explain EMDR Therapy treatment effects is called the AIP model. The AIP model, the model of pathogenesis and change in EMDR Therapy, was developed to explain the rapid change towards positive resolution that is observed in EMDR memory reprocessing (Shapiro, 2001). AIP assumes “an inherent system in all of us that is physiologically geared to process information to a state of mental health” (Shapiro, 2002). ‘‘Information’’ as it is used here, refers to external or internal input via all sensory systems, otherwise referred to as experience. In EMDR Therapy it is presumed that the neurophysiological activity of the AIP system in the brain leads to a reduction in distress and/or negative emotions that can be encoded^1^ as a result of upsetting experiences, leading to integration of upsetting information into a more adaptive, positive state. The AIP system may be hindered or blocked by trauma, severe levels of stress, or other variables like the influence of psychoactive drugs (Hase et al., 2008).

Shapiro stressed the fact that the client should be sufficiently resourced before the therapist will engage the client in memory reprocessing. In her textbook on EMDR Therapy 2nd edition, Shapiro (2001) states: “For many of our clients, it appears that simply reprocessing these earlier experiences allows the appropriate cognitive and emotional connections to be made and adaptive behaviors to spontaneously emerge, along with insights and positive self-concepts” (p. 5). However, for clients who have been badly neglected or abused in childhood, it is also important to determine what developmental windows might have closed before important infrastructures were set in place. Did the traumatized child learn object constancy, or will it need to be developed during therapy? What will the clinician have to model for the client? What experiences will have to be engendered both in and out of therapy to allow the needed introjects and patterns to emerge? As these positive interactions are forged within the therapeutic relationship, they are also stored in memory and can be enhanced by EMDR procedures. In the 3rd edition of her text, Shapiro (2018) again addresses these issues in a case example of a rape victim: “After her clinician has effectively treated her client for a violent rape experience using EMDR therapy procedures, the rape victim will be able to recall the rape without feelings of fear and shame….” On the following page, Shapiro states, “…Clinicians must understand how to prepare clients appropriately and stay attuned to their individual needs while keeping the information processing system activated so learning can take place.” (p. 3).

Clinicians must also take a comprehensive history to identify the appropriate targets for processing and the developmental deficits to be addressed. “In the current EMDR Basic Training Manual Part One (Shapiro and Laliotis, 2017) this is put as:” “Adaptive memory networks consist of associated memories that are processed and integrated. They include negative experiences that are resolved; i.e., the information is congruent with the emotional response and are no longer disturbing. It also includes positive life experiences (images, thoughts, feelings, physical sensations, and beliefs), that, when accessed, can be strengthened and enhanced through applying bilateral stimulation. Adaptive memory networks need to be present and accessible for reprocessing to occur. Therapeutic relationship is part of an adaptive memory network.” Explaining reprocessing, the manual states: “Accessing experiences (positive and negative) allows for the linkages between consciousness and where information is stored. Maladaptive/dysfunctional memory networks, when accessed and reprocessed, link with existing positive, adaptive memory networks.” The manual is very clear in instructions to the clinician: “Determine availability of positive/adaptive memory networks in order to proceed with memory processing. Develop and strengthen positive memory networks as needed.”

Shapiro obviously conceptualized the AIP Model not only as a model of the unprocessed but also as a model of positive experiences or resources, needed to reprocess the unprocessed. Interestingly Shapiro explicitly mentions the therapeutic relationship in her textbook and even more explicitly in the EMDR Basic Training Manual, but refrains from describing the therapeutic relationship in EMDR Therapy in more detail. The therapeutic relationship is an important component of EMDR Therapy but differs from the therapeutic relationship in other psychotherapeutic approaches. Dworkin (2005) introduced the relational perspective, but a description of the special therapeutic relationship in EMDR Therapy is still missing in the literature. It seems necessary to begin to elaborate this topic by describing the therapeutic relationship as one of the core elements of EMDR Therapy.

## A Hierarchy in EMDR Therapy

While the dynamic evolution of EMDR Therapy is offering more options to treat a variety of patients suffering from various disorders, the risk of misunderstandings increase. As research shows that following the treatment manual in EMDR Therapy is important to achieve positive treatment effects (Maxfield and Hyer, 2002), it’s also important to offer a larger framework that allows for clinical adaptations while adhering to the fundamental precepts of this model. This article proposes six levels or core elements of EMDR Therapy that are inherent to this comprehensive treatment approach regardless of the diagnosis, specialized protocols or context in which the treatment is being administered. Assuming a hierarchy in EMDR Therapy could contribute to more clarity. The first level is constitutive for the following levels, the second for the following and so forth. The therapeutic relationship should be added as another core element.

## The First Level: The Adaptive Information Processing Model

The cornerstone of this treatment approach is the AIP model, the model of pathogenesis and change within EMDR Therapy. This model proposes that our organism is naturally geared to move towards health in the same way that when we get a cut, our bodies respond in a way that promotes healing. When a negative experience overwhelms the information processing system of the brain, however, we are unable to process the experience to resolution and the memory is maladaptively encoded. These experiences that are unadequately processed and maladaptively encoded generate symptoms and become the focus of treatment. The AIP model guides our clinical actions from the first moments with a new patient until termination of the EMDR Therapy treatment. The AIP model informs our diagnostic procedures as well as our clinical actions. As the AIP model is an information processing model, it is important to have a comprehensive understanding of the client’s life experiences both positive and negative, and their access to adaptive information, often referred to as resources in EMDR Therapy. It is important to understand, that experiences which had a negative impact at the time of the event, may become a resource when they have been processed and are adaptively encoded. Understanding the client within the AIP model informs our understanding of client readiness to approach memory reprocessing, and the extent to which resource enhancement or development is indicated as preparation for memory reprocessing.

## The Second Level: The Eight Phases of EMDR Therapy

The second level in EMDR Therapy is constituted by the eight phases of EMDR Therapy. The eight phases describe the procedural steps as well as the course of treatment from the first moment to the last session. While they are referred to as phases, there are not equal or sequential, as the phases are distributed unevenly over the course of EMDR Therapy.

Phase1 (history taking) is the starting point of an EMDR Therapy treatment. Of course, Phase 1 activities are focused on gathering information contributing to an AIP informed case conceptualization and treatment plan, making determination about what memories and in which order will be targeted for reprocessing. Phase 1 is also the starting point for the emergent therapeutic relationship as well as the development of a therapeutic alliance. The stance of an EMDR therapist is non-judgmental, supportive, and understands the client’s clinical complaints as driven by inadequately processed memories that are being triggered by the client’s daily life demands. The ability of the therapist to attune to the client, and titrate the intensity of history taking to the clients affect tolerance contribute to success in Phase 1. With the typical PTSD or adjustment disordered client, Phase 1 may be limited to a few sessions, whereas the client who presents with a number of symptoms over a broad context, who may display dissociative symptoms or personality difficulties may be titrated over time. Typical Phase 1 procedures such as direct questioning (DQ), the Floatback technique (FB), or Affect Scan (AS) are applied for the purposes of mapping out the memory network of experiences informing the client’s current difficulties. In addition to these established procedures, history taking is enhanced by observing indicators of these inadequately processed and maladaptively encoded memories in their state-specific form. For example, a client who is coming for treatment due to severe anxiety becomes visibly overwhelmed while trying to relate their experience. To elaborate this briefly some reflections on a memory or node is necessary. The node in EMDR Therapy is the metaphor for the inadequately processed and maladaptively encoded memory, a memory network in itself as well as a portal into memory networks (Shapiro, 2018). The node could contain all information present at the moment of the event. This could be sensory information, thoughts, emotions, as well as perceptions about the present that are more informed by the past. Activation of a node generates symptoms in the present that we often refer to as being triggered. The past becomes present. In a psychodynamic therapy setting they would be addressed as transference. In EMDR Therapy transference is a symptom indicating activation of a node, which offers an invitation to explore the connection between the client’s current reaction and the earlier event(s) that are being activated.

Phase 2 (preparation/stabilization) is about determining client readiness for processing. The EMDR model is explained, and any concerns about the treatment are addressed. It also includes establishing the mechanics of how the treatment will be administered, to include the bilateral stimulation, seating positions, as well as a stop signal in case the client needs to stop during the processing itself. This is also the phase where resourcing interventions are offered as needed, even if the use of resourcing interventions is not limited to this phase and may be used over the whole course of treatment especially with the complex client. The number of Phase 2 sessions necessary to prepare the client for subsequent memory reprocessing differs greatly from client to client. A positive first experience with bilateral stimulation, as well as with the following first reprocessing sessions seem to facilitate a favorable outcome in the treatment of a PTSD patient in general (Lee, 2006).

Phase 3, Assessment, involves accessing the Target memory as it’s currently being experienced by the client and taking baseline measurements of the sensory components of their experience at the outset. During the reprocessing phases, Phase 4–6, the Target Memory and associated linkages to other memories are reprocessed. Fast bilateral stimulation is used to activate the client’s inherent information processing system for 20–30 s at a time with a brief check in to ensure that the process is moving. Byproducts of reprocessing such as insights, shifts in the client’s emotional response, a new, more adaptive understanding of what happend in the past, as well as assigning meaning to the experience are indicators of the process moving towards resolution. Phase 7, Closure, is designed to close down any session, espeacially a reprocessing session, whether it lead to complete or incomplete reprocessing of the target memory. Phase 8 is used to follow-up on every session. It takes place in the subsequent session where the therapist is asking for feedback on their experiences globally as well as evaluating the Target Memory itself.

## The Third Level: The Protocols

The sequence of memory reprocessing within the eight phases of EMDR therapy can be organized according to a certain protocol, a treatment plan. The protocols in EMDR Therapy help the therapist determine a particular sequence in the targeting of memories that are driving the client’s disturbance in the present. The Standard, Three-pronged protocol of EMDR Therapy outlines that past experiences are processed initially as they are driving the client’s current symptoms. Present triggers that have not been resolved by addressing the past experiences are addressed, followed up by a Future Template. The majority of published protocols follows the sequence of the standard protocol and ads some adaptations addressing the needs of a special population. The robust and well researched sequence of the standard protocol guaranties a safe and efficacious treatment for the majority of clients. Only the inverted standard protocol (Hofmann, 2009b) is reversing the sequence addressing the future first and slowly moving back into the past. This protocol is considered a safe alternative for the fragile, complex client. As any protocol is applied within the framework of the eight phases, it is to assume that preparatory and stabilizing work, Phase 2, is always part of the treatment, even if this is not mentioned in a certain protocol.

## The Fourth Level: The Procedures

The procedures in EMDR Therapy are manualized approaches to modify memories. These can be distinguished regarding the type of memory addressed, as resource installation procedures are applied to develop positive, resource memories (Leeds, 2009), facilitate the access to such memories or enhance resource memory networks. Till today a variety of resource installation procedures have been described. From the simple “Position of Power” by Popky, the more sophisticated “Resource Development and Installation” (Korn and Leeds, 2002) or more recent procedures like “Instant Resource Installation (IRI)” or “extended Resource Installation (xtRI)” (Hase, 2021), the variety seems to be able to meet the needs of different clients from childhood to serenity. Speed of bilateral stimulation (BLS) in resource installation procedures is in general slow. It has been shown, that the slow BLS lead to a different activity pattern in the brain, then fast BLS. The pattern induced by slow BLS is supposed to facilitate the access to positive memories (Amano and Toichi, 2016).

An other group of procedures aims to reprocess or at least modify the inadequately processed and maladaptively stored memories. Speed of stimulation with these procedures is in general fast, e.g., one movement per second. The reprocessing phases 4–6 constitute the core memory reprocessing procedure in EMDR Therapy with the ability for complete reprocessing of the memory and highest associative power, during the course of an EMDR Therapy treatment. Memory reprocessing procedures with reduced associative power could be considered EMD and the Four-Fields-Technique.

Another procedure will install the resource of present orientation, using slow BLS, before exposition in sensu to an unprocessed memory. The repeated to and fro between resource installation and exposition in sensu can lead to partial modification of the memory accompanied by symptom reduction or reduction in disturbance. This procedures is called CIPOS (Knipe, 2008). This procedure could be considered as a “Bridge Procedure,” bridging the gap between pure resource installation procedures and reprocessing procedures. CIPOS is often applied in early stages of therapy like reducing the impact of trigger experiences thus increasing client stability. It is important to keep in mind that the application of CIPOS does lead to complete memory reprocessing with the majority of cases. Recently the Flash Technique (Manfield et al., 2017) was introduced. It shows some similarities but needs further research and evaluation regarding the position in EMDR Therapy. Table 1 offers an overview on the procedures in EMDR Therapy regarding a description of quality.

**TABLE 1:** Procedures in EMDR therapy.

Description of Quality	Procedure
**Reprocessing procedures**	
High associative power	Phase 4–6
Low associative power	EMD, Four-fields
Bridging procedures	CIPOS
Resourcing procedures	PoP, RDI, IRI, xtRI…

The therapist will often opt for phases 4–6 reprocessing procedure as this is geared for complete memory reprocessing and strong association into the memory network. But considerating client stability, complexity or requirements of the session the therapist could opt for another procedure to initiate modification of memory. Beginning with a procedure leading only to symptom reduction, will more often lead to subsequent application of phase 4–6 procedure and complete reprocessing of target memory in subsequent sessions.

The compilation of procedures in this article is not comprehensive and may be outdated at the time of publication due to the speed of innovation in EMDR Therapy. Still it seems sufficient to outline principles.

The procedures in EMDR Therapy relate somehow to the Eight Phases. For instance diagnostic procedures like DQ, floatback or AS are used in Phase 1 and ressource installation often, but not exclusively, in Phase 2. Table 2 offers an overview on allocation of procedures to the eight Phases.

**TABLE 2:** Procedures in EMDR therapy related to the eight phases.

Phases	Procedures
Phase 1	Direct questioning, floatback, affect scan
Phase 2	Resourcing
Phase 7	Resourcing; floatback, affect scan
Phase 8	Direct questioning, floatback, affect scan

## The Fifth Level: Dual Attention Bilateral Stimulation

The alternating BLS, described by Shapiro as dual attention BLS, in memory reprocessing or memory modification procedures is of great importance. EM are considered the primary form of BLS, with the greatest impact on the information processing system. Alternating bilateral tactile stimulation or auditory stimulation are alternatives, which lack in impact as well as evidence in science. One has to consider, that the majority of clinical studies backing EMDR Therapy as an evidenced based treatment for PTSD were using EM. The appropriate dosage, speed and rhythm within resource installation and memory reprocessing is of great importance. If the therapist is able to monitor the clients process and adjust the BLS to the client’s needs, this contributes to the safe environment and the special therapeutic relationship in EMDR Therapy. This was described as neurophysiological empathy by Hofmann (2009a).

## The Sixt Element: Clinical Interventions

Clinical interventions in EMDR Therapy are used to facilitate processing if the client is blocked in processing or if the therapist wants to add tracks of information to optimize treatment effects. Blocked processing is defined as no change in the disturbing material after two consecutive sets of BLS. There are also many clinical situations where there is a reduction in distress but limited resolution, due to missing information and/or skills, and is in need of developmental repair for complete resolution of the Target Memory. These interventions are referred to as cognitive interweaves as they are clinician administered questions or comments designed to mimic reprocessing effects.

## Another Core Element: The Therapeutic Relationship

The importance of the therapeutic relationship has been well established in our profession. From the beginning, Shapiro refers to the importance of the therapeutic relationship in her many books and articles and even more explicitly in the EMDR Basic Training Manual, Parts 1 and 2 (Shapiro and Laliotis, 2017). Dworkin (2005) referred to the therapeutic relationship in his book on the “relational imperative” describing the centrality of the therapeutic relationship in EMDR Therapy from a psychodynamic perspective.

According to the American Psychological Association, the definition of evidence-based practice (ESB) both in the United States and internationally includes a combination of the best available research, the clinical expertise of the therapist, and patient characteristics (Norcross and Lambert, 2011). It stands to reason, therefore, that the therapeutic relationship is a core element of EMDR Therapy as in all psychotherapies. Research shows that the therapeutic relationship accounts for as much of the treatment outcome as the method itself (Norcross and Lambert, 2011). More specifically, the extent to which the therapist is able to accommodate specific client preferences related to culture, gender, race as well as religion and spiritually, the greater the likelihood of a positive treatment outcome (Swift et al., 2011). Moreover, when clients are asked to account for their success in psychotherapy, over 90% of respondents described their relationship with the therapist as primary (Norcross and Lambert, 2011).

Clients often come for psychotherapy for self-esteem issues, relationship problems and difficulties in self-regulation. These complaints are rooted often in attachment-based experiences that are formative in nature. It is understood that the process of psychotherapy activates the attachment system due to the nature of the therapeutic relationship. During memory processing for example, it’s not just about what happened to the client; it’s about what is happening in the moment to moment unfolding of experience in the therapy room. This requires the therapist to stay attuned to the client’s experience mostly through tracking nonverbal gestures, co-regulating the client’s experience and maintaining an empathic connection. EMDR therapists speak to their clients, but more often, it’s about being in the resonance with them (Siegel, 2010). We, as EMDR Therapists, keep eye contact, while at the same time being in conncection without being intrusive. Sometimes we even offer touch. Most importantly, we are in relationship to our clients and their experience in real time; we perceive our clients’ signals, maintaining a mindful presence, while responding promptly and appropriately to what is needed. The inherent processing demands of EMDR Therapy require a high level of safety and trust in the relationship as well as in the process itself, in order for most clients to be able to venture into the unchartered emotional territory of their past traumas. Flores (2013) highlighted the need for attachment as a lifelong need not limited to childhood, but as part of all relationships throughout the lifespan. The therapeutic relationship is no exception.

## Implications for Training, Consultation, Practice and Research

The development of EMDR Therapy from a desensitization technique to a comprehensive psychotherapy approach has many implications for training, case consultation, clinical practice and research. The development of protocols and procedures not only enrich EMDR Therapy, but make it more complex and sometimes confusing. Adhering to the basics of Shapiro’s model and training curriculum is fundamental for for the future of EMDR Therapy, yet at the same time, it will inevitably continue to grow and develop. Defining the core elements and structure of EMDR Therapy allows for innovations as well as new perspectives, such as a relational emphasis for attachment-based trauma, could enrich AIP-informed case conceptualization, treatment planning and even research.

From a training perspective, the established Basic Course curriculum should be understood as foundational. However, for most of us who have been teaching and practicing for many years, this methodology is easy to learn, but hard to do. It takes additional advanced trainings, ongoing case consultation well beyond the minimum standards, as well as personal therapy in order to fully understand and implement this approach.

Defining the a structure, six levels in EMDR Therapy, allows for innovation while staying true to the basic precepts and procedures of the model. In addition, it offers a structure that can inform clinical choice points.

These structures will also inform clearer guidelines for research. The reflections on the nature of the therapeutic relationship in EMDR therapy, for example, could spark new research, especially at a time where EMDR therapy in the treatment of attachment disorders is gaining momentum (Civilotti et al., 2019). The added emphasis on the therapeutic relationship as another core element and attachment theory (Brisch, 2015) could be an added incentive for the new trainee in to embrace the protocols and procedures, rather than seeing it as too mechanical and manualized. Perhaps an additional focus of the experiential component of trainings could include inviting the therapist to work on their own concerns about the developing role as an EMDR therapist. The reflections on the specific nature of the therapeutic relationship in EMDR Therapy and the therapist’s capacity to, go there’can be part of the training experience.

## Summary

The dynamic evolution of EMDR Therapy offers extraordinary opportunities for healing to clients suffering from various symptoms and problems. The AIP Model is ideal to gain understanding of a client’s pathogenesis in a non-pathologizing way and reach out to the suffering human being, offering a comprehensive approach, which can be tailored to the individual’s needs, whether it’s for symptom relief or for a more comprehensive treatment. The AIP model as a theoretical underpinning should be emphasized for case conceptualization as well as treatment planning, along with how to use the model informs how and when we make adaptations to best meet the needs of our clients.

Describing the structure in EMDR Therapy referring to six levels, with the AIP model constituting the uppermost level guiding case conceptualization as well as treatment in EMDR therapy, down to the clinical interventions in processing, could offer more clarity in teaching as well as research on applying EMDR Therapy protocols and procedures. Assigning importance to the therapeutic relationship as another core element in EMDR Therapy is necessary if we are going to refer to this methodology as a therapy that treats a broad range of clinical problems, and when we espouse ourselves to be an evidence-based approach. Relating the importance of the therapeutic relationship to attachment theory as part of training and practice is important for future training, case consultation and research, as many of us treat attachment-based disorders. Above all, as therapists we are all committed to best practices which requires us to go beyond the administration of a therapy method and accompany our clients on their journey towards healing.

## Data Availability Statement

The original contributions presented in the study are included in the article/supplementary material, further inquiries can be directed to the corresponding author/s.

## Author Contributions

The author confirms being the sole contributor of this work and has approved it for publication.

## Conflict of Interest

The author offers EMDR training and consultation.

## Footnotes

1 Shapiro used the term “stored.” As in science today the terminus technicus is “encoded” this will be used in the text.
